# To tax or to ban? A discrete choice experiment to elicit public preferences for phasing out glyphosate use in agriculture

**DOI:** 10.1371/journal.pone.0283131

**Published:** 2023-03-16

**Authors:** Amalie Bjørnåvold, Maia David, Vincent Mermet-Bijon, Olivier Beaumais, Romain Crastes dit Sourd, Steven Van Passel, Vincent Martinet

**Affiliations:** 1 Department of Engineering Management, Faculty of Business and Economics, University of Antwerp, Antwerp, Belgium; 2 Université Paris-Saclay, INRAE, AgroParisTech, Paris-Saclay Applied Economics, Palaiseau, France; 3 University of Rouen Normandy, LERN, Rouen, France; 4 Leeds University Business School, Leeds, United Kingdom; 5 Université Paris-Saclay, ENS Paris-Saclay, Centre d’Economie de l’ENS Paris-Saclay, Gif-sur-Yvette, France; Centre for Studies in Social Sciences, Calcutta, INDIA

## Abstract

In 2023, the European Union will vote on the reauthorization of glyphosate use, renewed in 2017 despite concern on impacts on the environment and public health. A ban is supported by several Member States but rejected by most farmers. What are citizens’ preferences to phase out glyphosate? To assess whether taxation could be an alternative to a ban, we conducted a discrete choice experiment in five European countries. Our results reveal that the general public is strongly willing to pay for a reduction in glyphosate use. However, while 75.5% of respondents stated to support a ban in the pre-experimental survey, experimental results reveal that in 73.35% of cases, earmarked taxation schemes are preferred when they lead to a strong reduction in glyphosate use for an increase in food price lower than that induced by a ban. When glyphosate reduction is balanced against its costs, a tax may be preferred.

## Introduction

Glyphosate—one of the world’s most widely used active substances in plant protection products—has in recent years become the subject of controversy over its safety and impact on the environment [1]. The European Union (EU) renewed its approval of the substance from 2018 to 2022 (and then until the end of 2023), despite experts and agencies warning of glyphosate’s carcinogenicity and its threat to ecosystems and biodiversity [2]. This decision arose against the backdrop of a European Citizens Initiative calling for its ban, which garnered 1.3 million signatures [3]. At the same time, many farmers and farmers’ unions are vigorously opposed to the ban, claiming that there are no economically viable alternatives for the chemical, and that a quick transition to farming without the use of glyphosate is too costly and difficult. According to an IPSOS-AgriAvis pool led in 2017, 81% of French farmers are against the ban—arguing that glyphosate is necessary for production, estimating that reducing glyphosate use would increase their production costs by 24% and reduce yield up by to 25%, and 57% of farmers state that it would be more relevant to limit its use rather than to ban it [4]. Despite continuous policy efforts to reduce the use of pesticides across the EU, their use has remained stagnant, and in some cases increased [5]. A new vote, initially scheduled in 2022, will take place at the end of 2023. The case for entirely phasing out the use of glyphosate across the EU and related controversies demonstrate the need for a broader dialogue between regulators, farmers and consumers on the options, alternatives and regulatory directions available to us [6].

Designing an optimal policy for glyphosate use regulation is not an easy task. Economic theory states that a polluting activity should be regulated at a level trading-off its social costs and benefits, i.e., at a level such that the marginal benefit of the activity (often private) equals its marginal (social) cost. If the costs overcome the benefits for any pollution level, the optimal pollution level is nil, and this optimum can be reached either through a ban or through a very large tax level. Otherwise, some pollution is socially beneficial, and the optimal pollution level can be reached either by setting a tax at a level equal to the marginal social cost of pollution (regulation through prices) or by allocating quotas among polluters (regulation through quantities) [7, 8]. In the case of glyphosate, given the uncertainty on the negative externalities it generates and the controversy on its use, the issue is not that much to determine an optimal use level and the corresponding optimal policy but to define an acceptable policy. In our study, we do not try to determine an optimal phasing-out policy, but discuss the acceptability of different policy options. As such, the preferred policy alternative from consumers’ perspectives may not correspond to the social optimum, as their preferences do not encompass all the implications (positive or negative) of glyphosate use.

Is a tax resulting in a strong reduction in glyphosate use a socially acceptable alternative to a ban? Environmental taxation is popular among economists for reasons of efficiency and environmental effectiveness [9]. In contrast to an outright ban, taxes allow regulators to influence application rates in a continuous (rather than all-or-nothing) manner, allowing fine-tuning at the farm scale and a progressive phasing-out. A tax is also an alternative to possible exemptions to a ban, which could lead to prolonged public and political debates and lawsuits (or even active lobbying) to determine who should and who should not use glyphosate. Taxation solves this on economic grounds. With a tax, farmers have the choice to continue using pesticides in certain high-value and extreme cases, while discouraging low value applications when alternatives exist.

Environmental taxation is, however, very unpopular among the general public [10]. The effectiveness of environmental taxes can be underestimated, and they can be perceived as coercive and less likely to drive a voluntary change in behavior than subsidies [11]. For fossil fuels, the unpopularity of environmental taxation has hindered their full implementation in Europe, where they have often been considered unfair, which sparked the Yellow Vest uprising in France for example [12], and led to exemptions in Scandinavian countries by fear of consequences on competitiveness, which decreased the potential for any considerable positive impact on the environment [13].

Introducing a tax on a market imposes a burden on consumers and producers. In theory, however, when implementing an environmental tax at the optimal level, the losses for producers and consumers are compensated for by gains to society due to the reduction in the environmental and health externalities of pollution. If the primary aim of pesticides taxes is to reduce their use cost-effectively, in the process these taxes also generate revenue. Using tax revenues to reduce income tax or value-added tax has been seen to be relatively unpopular [12]. People are more willing to accept them if tax revenues are used to strengthen their environmental effectiveness [14, 15]. Earmarking is therefore popular, as taxpayers might consider that tax revenues are otherwise used wastefully or for something they disapprove of [16] and earmarking can then overcome distrust in government regarding ecological tax reform [17]. In the case of a pesticide tax, it would also be able to compensate farmers for lower production and indirectly lead to price-driven shifts to sustainable agriculture [9].

Even though pesticide taxes could contribute to better policies, they are rarely used in the current policy mix. While many studies have shown that high tax rates should be applied to attain desirable reductions in the use of pesticides [18–20], pesticide taxes currently only exist at very low rates in Denmark, Sweden, Norway, Italy and France, and at least in the latter four, these taxes are so low that they have a negligible impact on the use of pesticides. Even if pesticide taxes are more allocatively efficient than many other policy instruments, such as bans or regulation, stakeholders’ preconceptions, preferences and concerns with regard to pesticide taxes have hindered further implementation [20, 21]. Understanding public opinion of such a policy measure among consumers has yet to be studied to ensure social acceptance of pesticide policy measures and to study the trade-offs underlying citizens’ preferences for public policy options to phase out glyphosate.

It is impossible to be certain that an EU-wide glyphosate ban will happen in 2023, but there is a public and political willingness within Europe to significantly reduce the use of glyphosate. The aim of our study is to test the acceptability of various policies, assuming that governments wish to drastically reduce the use of glyphosate. Understanding how to implement an effective pesticide tax regime to phase out the use of glyphosate requires balancing political feasibility and public acceptance considerations in line with tax and environmental policy. While the rationale for a tax on glyphosate lies in the abatement incentives and reduction of pesticides that it might create among farmers, the question arises as to how the use of revenue from pesticide pricing affects its political feasibility–and specifically the preferences of the consumers of the agricultural products whose prices will likely be impacted. To help answer this question, we conducted a Discrete Choice Experiment (DCE) with 2,050 individuals from a representative sample of the population in five European countries: Belgium, France, Germany, Italy and Spain.

## Results

### Discrete Choice Experiment (DCE)

A DCE [22] is a useful approach to investigate preferences for alternatives when no data is available from observed behavior, which is the case when one considers prospective, future policies. It presents a set of hypothetical choice tasks to respondents, who are asked to choose a favorite among several alternatives within each task. Often, three alternatives are presented: a reference alternative which is the same in all choice tasks and can represent a status quo or opt-out option, and two other alternatives which vary among choice tasks. Alternatives are made up of different attributes, i.e., fundamental characteristics of the scenario, and each attribute can take different levels. When one of the attributes is a price or a cost, the method makes it possible to elicit the willingness-to-pay (WTP) or willingness-to-accept (WTA) for changes in the other attributes’ level, i.e., the monetary equivalent of a change in the level of a non-monetary attribute. This feature makes DCE a useful method to estimate preferences for goods or amenities which do not have a market price, such as health or the environment [23].

DCEs have been used to investigate preferences on pesticide reduction measures, showing consumers’ significant willingness-to-pay for the reduction of pesticide use for health and environmental reasons [24–26]. Farmers too value the benefits of reducing pesticide use [27], but are averse to the risk of substantial losses in production implied by the adoption of low-pesticide practices [28, 29]. Regarding glyphosate use, the DCE approach has also been used to show that farmers prefer the use of glyphosate to other alternatives to prevent weed infestation, because it saves work and labor costs, especially on large farms [30].

**Fig 1:**
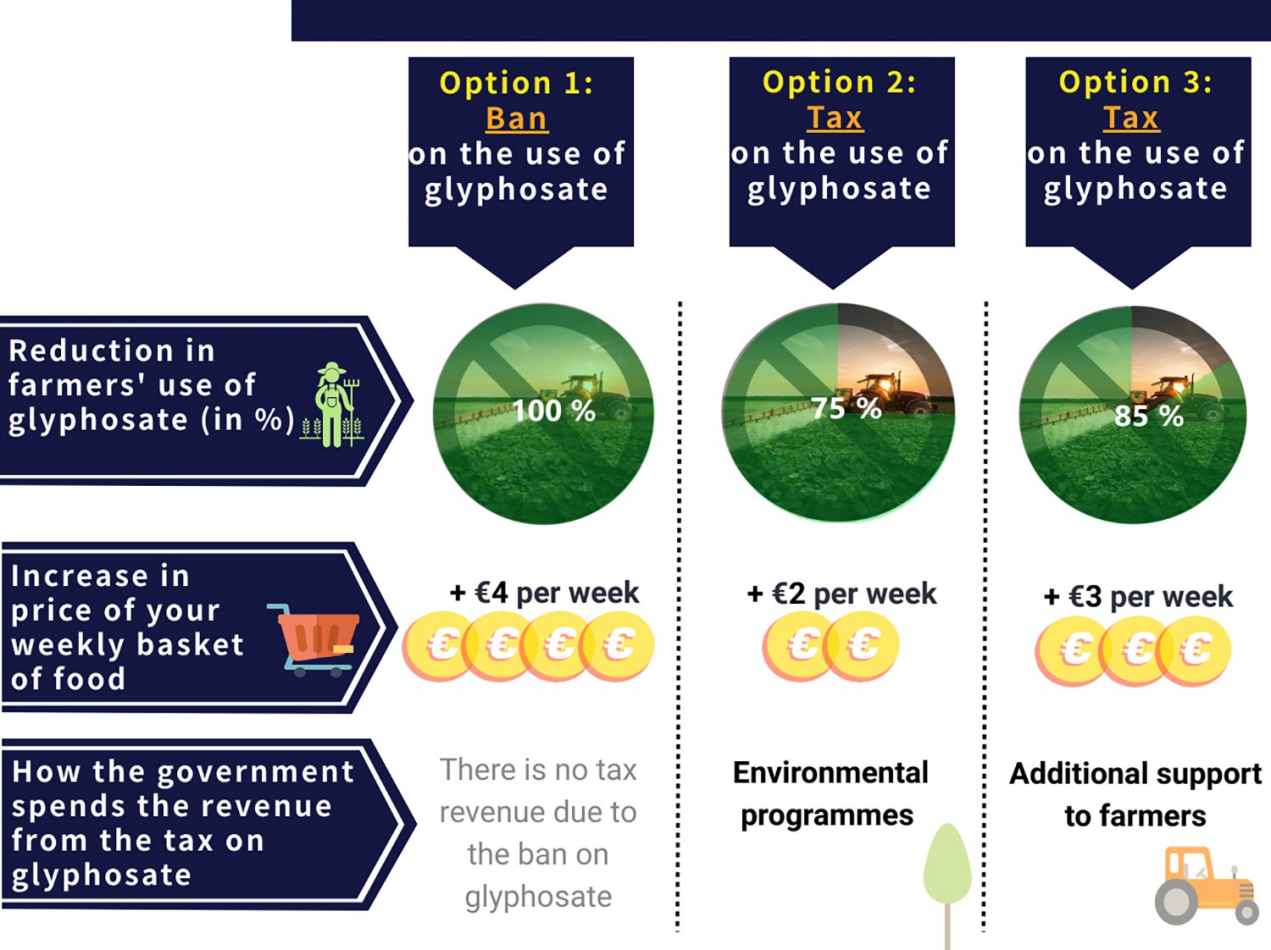
Example of a choice task (English translation). Compared to the ban (reference policy) both tax alternatives result in lower reduction in farmers’ use of glyphosate (respectively of 75% for option 2 and 85% for option 3, to be compared with the 100% reduction of the ban) but have a lower cost for consumers (respectively +€2 per week for option 2 and +€3 per week for option 3, to be compared with the +€4 per week for the ban). The two tax alternatives also differ in the way the government spends the revenue from the tax (respectively for environmental programs for option 2 and for an additional support to farmers’ transition for option 3). The attribute levels are different in the other choice tasks. A full description of the 12 choice tasks is provided in S1 Table in the S1 File.

Our DCE investigates the preferences of the general population in five European countries–Belgium, France, Germany, Italy and Spain–regarding policy instruments aiming to drastically reduce the use of glyphosate in agriculture. Respondents were faced with six sequential choice tasks, each asking them to choose their preferred policy option among three alternatives: a reference scenario corresponding to a total ban of glyphosate, judged to be the most probable policy, and two taxation scenarios which vary over the choice tasks. An example of a choice task is depicted in Fig 1. S1 File detail the method and provide justifications of the choices we operated to design the experiment.

Each policy option is described by three attributes, which can take different values (levels) summarized in Table 1. The first attribute refers to the reduction of farmers’ use of glyphosate in agriculture, of 100% for a ban and ranging from to 75% to 95% for a tax. The second attribute refers to the increase in price of a respondent’s weekly grocery basket of food, corresponding to 4 euros for a ban and ranging from 1 to 3 euros for a tax. The third attribute corresponds to a possible earmark of the tax revenue, capturing the preferences of respondents on how the revenue from a hypothetical tax on glyphosate would be spent. This attribute was balanced against the flow of tax revenue to the government budget to programs rather dedicated to one of the main issues relevant to a partial reduction of glyphosate use by farmers: effect on public health, effect on the environment and the likely loss of revenue for farmers on their way to transitioning to more sustainable forms of agriculture [30–32]. The respondents were provided with a full description of the attributes and their levels prior to completing the discrete choice experiment.

**Table 1:** Attributes and levels of the Discrete Choice Experiment.

Attribute	Attribute levels
**Reduction in farmers’ use of glyphosate (in %)**	75%; 85%; 95%; 100% (**Ref**)
Increase in price of weekly shopping basket of food	€1; €2; €3; €4 (**Ref**)
How the government spends the revenue from the tax on glyphosate	Environmental programs;
	Health programs;
	Additional support for farmers;
	Revenue flown into the government’s general budget;
	No tax revenue (**Ref**)

**Ref**: *only possible in the reference situation (ban)*

Two sets of six choice tasks were experimentally designed using the Ngene software so that the respondents’ choices provide the maximum information regarding their preferences over the attributes and levels. All choice tasks were rationally consistent, with lower levels of reduction in farmers’ use of glyphosate being associated with lower increase in price of weekly shopping basket of food. S1 Table in the S1 File provides a full description of the 12 choice tasks and the way they were grouped in the two choice sets of 6 choice tasks.

Our sample of respondents consists of 2050 households’ primary grocery shopper, aged 18–70 years-old. Respondents were recruited by the survey agency IPSOS, which abides by the ICC/ESOMAR International Code on Market and Social Research regarding ethics in social sciences research. Each country’s sample is representative of the national population at the household level on the following criteria: age and gender of the primary grocery shopper, geographical location (region; rural vs. urban) and level of education. Each respondent completed one of the sets of choice tasks (randomly assigned, but maintaining the representativeness for the two sub-groups), so that we obtain 2050 x 6 = 12300 choice observations.

Prior to completing the discrete choice experiment, the respondents filled in a survey to gather information on their personal situation, i.e., the number of people living in their household and their approximate spending on food per week. These factual data, along with the socio-economic information used for representativeness, were used in the econometric analysis. The survey also asked how often they purchased organic food and whether they had ever heard of glyphosate. Respondents that had not heard of glyphosate were kept in the sample, as they will be equally impacted by a price increase due to a regulation of glyphosate use. Respondents were also asked about their views on the effect of glyphosate/pesticides (whether they knew glyphosate or not) on the environment and health, and whether they agreed with a ban or a tax on glyphosate/pesticides. Relating to the contents of the DCE, respondents ranked options for how their government should spend hypothetical tax revenue from agricultural use of pesticides among environmental programs, health programs, additional support for farmers, or flow into the government’s budget for unspecified use. Such attitudinal information was used for consistency checks and results discussion, but not for the econometric analysis, to avoid endogeneity issues.

### Quantifying respondents’ preferences

We analyzed the respondents’ choices with a Latent Class Model with two classes. The statistical analysis is described in the Supplementary text in S1 File, providing a full description of this model. This model is useful to take into account respondents’ heterogeneity and the existence of classes of respondents, which was particularly relevant in our case study, which aims at assessing pros and cons to a glyphosate ban. The model allocates (probabilistically) respondents to one of two groups, depending on their choices, and characterizes the preferences of each group as well as the variables that influence the probability of belonging to a group (Table 2). Table 2 presents the coefficients associated with each attribute for the two classes, as well as the coefficients associated with the non-reference situations (ASC2 and ASC3), i.e., the tax alternatives. More precisely, these ASC2 and ASC3 coefficients indicate how the respondents’ utility varies when being in the tax situation compared to the ban situation. A positive value reveals a preference for the tax, *ceteris paribus*, whereas a negative value corresponds to a preference for the ban. The respondents allocated to class A exhibit a significant preference for tax alternatives (positive coefficients for the variables ASC2 and ASC3), whereas the respondents allocated to class B strongly reject any alternative to the ban option (strongly negative and significant coefficients for the variables ASC2 and ASC3). We thus interpret these classes as anti-ban (A) and pro-ban (B). In both classes, the price coefficient is strongly significant and negative: all respondents prefer a policy option with a lower financial cost. This negative effect of price is, however, highly heterogeneous, as the standard deviation of the distribution is large and significant in both classes. Also, the respondents value the reduction of glyphosate use by farmers (strongly significant and positive coefficients for “Reduction in farmers’ use”), but pro-ban respondents value it much more than anti-ban respondents. Last, the respondents in the two classes value the fact that a policy earmarks the revenue of the tax to specific programs rather than flowing in the government’s general budget (strongly significant and positive coefficients for “Environmental programs”, “Health programs” and “Additional support for farmers”), with a preference for the support to farmers.

**Table 2:** Latent Class Model.

Results of latent-class model		
	*Class A*	*Class B*
Price	-2.045***	-2.641***
	(0.132)	(0.307)
Price: standard-deviation (σ)	1.915***	1.636***
	(0.114)	(0.184)
Reduction in farmers’ use	0.030***	0.067***
	(0.004)	(0.012)
Environmental programs	0.968***	0.821***
	(0.056)	(0.248)
Health programs	0.991***	0.559***
	(0.056)	(0.212)
Additional support for farmers	1.407***	1.151***
	(0.067)	(0.221)
ASC2	0.415***	-3.305***
	(0.082)	(0.240)
ASC3	0.161*	-3.913***
	(0.088)	(0.261)
*Class A*: *Allocation*		
Constant	2.999***	
	(0.375)	
Age	-0.021***	
	(0.004)	
Female	0.037	
	(0.100)	
Household size	0.090*	
	(0.050)	
Education	-0.087	
	(0.068)	
Rural	-0.257**	
	(0.126)	
Food expenditures	-0.004***	
	(0.001)	
France	-1.094***	
	(0.260)	
Germany	-1.206***	
	(0.256)	
Italy	-1.168***	
	(0.259)	
Spain	-1.038***	
	(0.268)	
Probability of allocation to Class A	64.15	
Probability of allocation to Class B	35.85	
Observations	12300	
Number of individuals	2050	
Log-likelihood	-9436.9	
Draws	10000	

Robust standard errors in parentheses

*** p<0.01

** p<0.05

* p<0.1

From this model, it is possible to assess the monetary value of non-monetary attributes, i.e., the amount needed for the respondent to accept to “reduce” the level of an attribute (named the willingness-to-accept, WTA). Here, it corresponds to the reduction in the cost supported by consumers which is needed to offset a one percent decrease of glyphosate use reduction (i.e., an additional percent of residual use) or the suppression of tax revenue’s earmarking. These results are displayed in Table 3. The two classes yield significantly different WTA measures only for the attribute labeled as “reduction in farmer’s use” (p = 0.007). The difference in the WTA for the other attributes is not statistically significant. Respondents in the pro-ban class are willing to accept residual glyphosate use only if it results in a reduction of the consumers’ cost of around €0.48 per percent. The respondents in the anti-ban class value each percentage point of residual glyphosate use at only €0.11, meaning that they are more likely to accept a policy with some residual glyphosate use at a milder economic cost. For both classes, earmarking is valued positively, between 2 and 7 euros, meaning that, above all, respondents strongly dislike tax policies without tax revenue recycling earmarks. The main difference in the two classes is thus the value granted to glyphosate reduction. The key issue is thus to understand what drives the allocation to these two classes.

**Table 3:** Marginal Willingness-To-Accept estimates, Krinsky & Robb.

Marginal Willingness-To-Accept estimates, Krinsky & Robb		
	Class A	Class B
Reduction in Farmers’ use	0.1138 (0.0929; 0.1347)	0.4801 (0.2507; 0.7094)
Environmental programs	3.7647 (2.7406; 4.7887)	6.0458 (0.8023; 11.2894)
Health programs	3.8572 (2.7999; 4.9146)	4.0883 (0.1469; 8.0298)
Additional support for farmers	5.4748 (4.0073; 6.9424)	8.4960 (2.1810; 14.8111)

Notes: The marginal willingness-to-accept is the monetary amount the respondents require to forgo an option (e.g., a percentage point of glyphosate use reduction).

95% confidence intervals are in parentheses. The Krinsky & Robb method is conducted with 100,000 draws.

Given that class allocation is probabilistic, it is not possible to know in which class a given respondent would be allocated with absolute certainty. However, it is possible to assess the probability to choose an alternative to the ban given the likelihood to belong to each class. Respondents chose a tax scenario in 73.35% of the choice tasks. For respondents whose likelihood to belong to the pro-ban class is above 50%, the probability of choosing an alternative other than the ban is 37.54%. When the likelihood of being pro-ban increases to 70%, the probability to choose an alternative to the ban decreases to 29.19%. Finally, when this likelihood is above 90%, the probability to choose an alternative to the ban goes down to 8.03%.

More than a third of our sample (35.85%) is statistically allocated to the pro-ban class, and the remaining 64.15% to the anti-ban class (see the second part of Table 2 for the detailed results on class allocation and its drivers; the elements in the next paragraphs are supported by these results). This allocation is significantly influenced by some socio-demographic characteristics of respondents, and the composition of the two classes differs: older respondents, respondents who spend more on their food, and rural households are more likely to be allocated to the pro-ban class.

Age is positively correlated with the awareness of glyphosate and the belief that glyphosate affects health and the environment. This suggests that older persons are more worried about the potential health impacts and environmental effects of glyphosate.

Regarding food expenditures, we interpret this result as an income effect: households could spend more on food because the household size is larger and/or because they spend more per capita, e.g., because they buy organic food. There is a positive and significant link between the size of the household, the frequency of organic food consumption and food expenditure. However, larger households are more likely to belong to the anti-ban class (at a 10% confidence), likely due to budget constraints and higher sensitivity to food price. This suggests that the food expenditure effect corresponds to households who spend more on food per capita and buy more organic food, and thus are concerned with what they eat.

As age is not correlated with greater food expenditure, and is negatively correlated with household size and with the consumption of organic food, these drivers in our data analysis are independent. A link between the two variables could be the respondents’ income, which we do not control for directly. Older respondents and households with larger food expenditures may have higher revenues which, according to the literature [33], would increase their probability of being sensitive to environmental issues. There is, however, no link between the preferences we study and the level of education, which is a less sensitive proxy for socioeconomic position than self-reported income.

Respondents who live in rural areas are also more likely to be pro-ban. In the pre-experimental survey data, rural households are more likely than the general population to believe that glyphosate affects health, but are not more likely to believe that it affects the environment. Their relative preference for a ban might be due to risk of exposure to glyphosate. These households have a preference for the earmarking of tax revenues to farmers’ support, though, emphasizing a concern for the consequences of glyphosate policies for farmers. Last, Belgian respondents are significantly less likely to be allocated to the pro-ban class than the other nationalities. Exogenous factors could explain this result, but none of the factual or attitudinal information from the pre-experiment survey explain this difference.

## Discussion

The question of phasing out glyphosate is highly controversial. Our study emphasizes the fact that statements on environmental policies taken in isolation with regard to the consequences of the considered policy may not fully encompass the multiple drivers of choices and the underlying trade-offs. Whereas the responses to our pre-experimental survey suggest that the general population favors a ban, with over 75% of respondents supporting the ban, our analysis of the experimental data unexpectedly reveals that, when balanced with the cost of the given policy, respondents prefer a tax that strongly reduces the use but at a moderate cost in the majority of cases, especially if the tax revenue is earmarked to specific programs to support farmers’ transition, or to protect public health or the environment. There is thus a trade-off between glyphosate use reduction and the cost of the policy for consumers. We analyzed the drivers of these trade-offs and quantified them using econometric techniques. Given the large size of our sample and high computer power, we obtain strongly significant and robust results.

Our results reveal that the general population is split regarding preferences on public policies to trigger the reduction of glyphosate use by farmers. On the one hand, a third of our sample seems to favor a ban at “any cost”, in the sense that they are not willing to accept residual glyphosate use against the proposed reduction in the cost of the policy. On the other hand, two-thirds of the sampled population is either against a ban or ready to accept residual glyphosate use if it decreases the cost of the policy sufficiently. Our Discrete Choice Experiment makes it possible to quantify these trade-offs. A deviation from the ban policy is costly to all respondents, but it is four times costlier for members of the pro-ban class (11 Euro cents per week per percentage point of residual use for those who do not favor a ban to up to 48 Euro cents per week per percentage for the pro-ban class). The general population is thus strongly favorable to a policy that results in massive reduction of glyphosate use by farmers.

A taxation policy has some advantages and limits. A tax puts an upper limit on the cost of reducing the use of glyphosate, whereas a ban would correspond to an “infinite” tax, meaning that glyphosate use has to be stopped whatever the cost for farmers and the society. When there are no alternatives to glyphosate at a tolerable cost, a ban may not be acceptable. A tax at a sufficiently high rate can accommodate a transition by phasing out most of the glyphosate uses, i.e., uses that have alternatives at a viable cost and thus a reduced impact on food price, while allowing a residual use for highly valuable purposes, i.e., for farmers facing alternatives having a higher cost than the tax, which could make the production cost skyrocket, or jeopardizing a production system. In France, there are mainly three types of production systems that rely on glyphosate without economically viable alternatives: soil conservation agriculture, for which tillage is not an option; seed (breeding) and plain field vegetable production, which cannot afford a contamination by weeds for purity or food safety reasons; few high-value niche production (e.g., flax, nuts) [34]. These producers may be willing to pay a lot to be able to use glyphosate pointwise.

A majority of respondents may be ready to accept such a milder policy if it reduces the cost for consumers sufficiently. In that perspective, the challenge is to set the tax at a proper level, consistent with the policy goal. This can be done progressively, by increasing the tax gradually to allow a smooth adjustment. Note that, as pesticide use is poorly elastic to pesticide cost (i.e., the use does not decrease a lot when the cost increases), the level of taxation we are discussing here is very high. The assessment of the cost of alternatives to glyphosate in France shows that, for field crops, in which the cost of glyphosate purchase was between 2 to 5 euros in 2020, the extra cost of substituting glyphosate by mechanical work is lower than €10 per ha and per year for about 80% of the cultivated areas, and larger than €25/ha/year for the other 20%, among which less than 2% are faced with a cost close to €80/ha [35]. This cost is larger than €100 in most vineyards, and about €500 in Alsace and Champagne (with a cost of glyphosate ranging from 15 to €65/ha) [36]. For orchards, the best alternative to glyphosate is to settle grass strips, at an extra cost around €150 to €200/ha (with an average cost of glyphosate lower than €10/ha) [37]. Multiplying glyphosate price by five to ten thus seems necessary to incentivize alternatives in most cases, while offering the option to keep using it in few sensitive cases. For orchards, such a level of taxation could be ineffective, but combining a tax with a subsidy to grass stripes could foster their adoption in place of glyphosate use.

Also, a ban is likely to come with (temporary) exemptions. Setting exceptions is controversial and potentially unfair, and could raise debates on the allowances; a tax treats every farm system the same. Moreover, a production with an exceptional allowance has no incentive to reduce use. With a tax, even farmers that prefer to pay the tax rather than to stop using glyphosate have an incentive to reduce use and search for alternatives. Our results also show that earmarking mitigates the rejection of environmental taxation.

Last, our analysis emphasizes that, if a referendum was run to settle the glyphosate question, the information provided on the consequences of the policy on food price on the one hand, and on the use of a possible tax revenue on the other hand, would strongly influence the results, given the importance of these drivers in the trade-offs studied in our Discrete Choice Experiment.

## Supporting information

S1 File (DOCX)
