# Supplementary material for: To tax or to ban? A discrete choice experiment to elicit public preferences for phasing out glyphosate use in agriculture
Source: PLoS One. 2023 Mar 16;18(3):e0283131. doi: 10.1371/journal.pone.0283131 (PMC10019652; doi:10.1371/journal.pone.0283131)
Supplement: S1 File — (DOCX) [file pone.0283131.s001.docx]

Supplementary Material for

**To tax or to ban? A Discrete Choice Experiment to elicit public preferences for phasing out glyphosate use in agriculture**

Amalie Bjørnåvold, Maïa David, Vincent Mermet-Bijon, Olivier Beaumais, Romain Crastes dit Sourd, Steven Van Passel, and Vincent Martinet*

*Corresponding author. Email: [vincent.martinet@inrae.fr](mailto:vincent.martinet@inrae.fr)

**This PDF file includes:**

Supplementary Text

Tables S1 to S3

References (38 to 59)

**Supplementary Text**

Discrete Choice Experiment: details

*On the choice of the countries included in the experiment*

The chosen countries include the four most populated countries in the EU, representing over 50% of the EU population (Eurostat, 2021), as well as Belgium. This choice is based on the fact that France is one of the main European producers of agricultural products and users of pesticides. The five main countries to which France exports these products are Germany, Belgium, Italy, the United Kingdom (UK) and Spain [^[[1]](#endnote-1)^]. The UK was excluded from the study due to a different currency and the fact that it is not part of the EU (and thus not concerned by the glyphosate authorization EU policy). The main terrestrial borders of metropolitan France are, by decreasing order, Spain, Belgium, Switzerland, Italy and Germany. Switzerland was excluded from the study due to a different currency and the fact that it is not part of the EU. The selected countries thus have an interest in the policies put in place to restrict the use of glyphosate for environmental or health purposes, either at the EU level or at the French level.

*On the choice of the ban as a reference policy scenario*

In the DCE literature, the benchmark option is not necessarily the current situation (status quo) or an opt-out option: choice tasks can present a future situation and the reference alternative is then the most probable or the business-as-usual future situation [^[[2]](#endnote-2)^,^[[3]](#endnote-3)^,^[[4]](#endnote-4)^]. To determine the reference scenario, we had to decide what the most probable policy outcome with regard to the future of agricultural glyphosate use would be in 2022 (as presented to the respondents), following the political trend at the time of the survey design. In November 2019, the French government announced that glyphosate would be banned from July 2021 onwards (which did not occur). In Germany, a ban is planned for 2023 - whatever happens regarding the EU vote on the glyphosate renewal. Even the Spanish government - which had voted in favor of a glyphosate renewal in 2017 - announced in May 2019 that it was considering a glyphosate ban. A study on glyphosate use in Spain was published based on 2015-2019 data provided by and funded by the Spanish Ministry for Ecological Transition [^[[5]](#endnote-5)^]. The results found that Spanish surface and groundwater samples were contaminated by concentrations of glyphosate hundreds of times higher than the legal limit values; these results could influence the Spanish vote.

Moreover, in 2017 when the approval for glyphosate was renewed at the EU level for the period 2018-2022, the vote was very close, and Italy, Belgium and France were among the nine countries opposed to this renewal. The German agricultural minister changed his stance at the last minute, from abstaining to supporting the proposal of extension, against the advice of the government. This sparked an uproar, and given the last German announcement, it can be assumed that Germany will not be among the bloc of countries voting for a renewal after 2022. Additionally, the UK - together with the Netherlands - led the coalition in favor of the authorization of glyphosate. Now that the UK is no longer a member of the EU, the balance of power between countries voting for and against an authorization will be significantly altered.

In 2017, the glyphosate re-authorization process took a different turn to what had previously been the case for pesticide re-licensing procedures, where decision-makers generally voted quietly in EU-committee meetings, without much public attention. In 2017, however, policymakers were faced with backlash from both the general public as well as civil society towards the renewal of glyphosate. Public concern had in particular been sparked as the World Health Organization had declared that glyphosate was a substance that “probably causes cancer in humans” [^[[6]](#endnote-6)^]. On the other hand, the European Chemicals Agency (ECHA) published an opinion in July 2022 stating that glyphosate does not cause cancer in humans, and finds that "the available scientific evidence did not meet the criteria to classify glyphosate for specific target organ toxicity, or as a carcinogenic, mutagenic or reprotoxic substance." ECHA did not classify glyphosate as carcinogenic in 2017 either, but still highlights that glyphosate can cause serious eye damage and is toxic to aquatic life. Farmers’ associations and interest groups representing the agrochemical industry simultaneously downplayed risks and stressed benefits of the substance in 2017. We can expect a similar turn of events in the upcoming vote, with continued lobbying activities from the Glyphosate Renewal Group - a group of eight agrochemical companies led by Bayer - seeking renewal of the approval of glyphosate use. A strengthened public interest can also be expected, perhaps due to a pronounced focus on public health as a result of the COVID-19 pandemic. While this is conjecture, another factor to consider was the finding that EU regulators had based their decision to re-license glyphosate on an assessment that had been plagiarized from industry reports [^[[7]](#endnote-7)^]. These factors will most likely influence policymakers’ decision-making and the likelihood of an imposed ban on glyphosate. Given the political context regarding glyphosate in the five countries we consider, we concluded that a ban on glyphosate was the most realistic business-as-usual option.

*On the choice of the attributes of the policy options*

The complexity of DCEs increases with the number of attributes and levels chosen [^[[8]](#endnote-8)^], which should be small to limit the cognitive burden imposed on respondents [^[[9]](#endnote-9)^]. The attributes and levels characterizing policy scenarios were refined from discussions with experts (scientists, agronomists, researchers, policymakers and farmers). Pre-tests and pilots being necessary to test the appropriateness of the attributes, select appropriate choice vehicles and to refine the draft questionnaire, we conducted several pre-tests with the authors' colleagues and acquaintances, as well as a pilot survey with the target population, tested via Amazon Mechanical Turk (M-Turk) - a crowd-sourcing website used to hire individuals to perform discrete on-demand tasks. Forty-five individuals (unknown to the authors) from the general population in Belgium, France, Germany, Italy and Spain completed the pre-test in their respective languages (nine from each country). Changes to the survey were iteratively made based on all comments and feedback received in each step of the testing process. We ultimately limited our description of policy scenarios to three attributes: the reduction in glyphosate use, the weekly cost, and the earmarking of the tax revenue.

As we aim at studying policy scenarios that go further than current policies, which look to reductions up to 50% [^[[10]](#endnote-10)^], we considered glyphosate reduction levels from 75% and above. Three possible levels for glyphosate reductions are considered for the taxation scenarios (-75%, -85% and -95%), whereas the ban is the only policy scenario achieving a -100% reduction.

Banning glyphosate would have a cost for farmers (at least in the short term, as this cost can decrease in the long-run according to the Porter hypothesis [^[[11]](#endnote-11)^]). INRAE estimated the consequences of the end of glyphosate for different farming systems. For main field crops, this cost ranges from 1% to 23.4% of the gross operating surplus per ha, depending on the current strategy to control weeds. The cost is low for farmers who already use tillage in addition to glyphosate, whereas it is huge for farmers that do not work the soil and saw directly after glyphosate use [35]. For arboriculture, the increase in weed control cost ranges from 6% to 20% of the gross operating surplus per ha (mainly varying with the fruit type) [37]. For viticulture, from 1% to 11.5% of the gross operating surplus per ha (mainly depending on the region, the slope of the plot, etc.) [36]. On January 30, 2019, the French Minister of Agriculture and Food, Didier Guillaume, claimed in an interview to France Info that the policies in place to increase sustainability of the agricultural sector and a reduction in the use of glyphosate could lead to an increase in the price of consumers' weekly baskets of food of 4 to 10%. The average spending on food (across all five countries) for a single-earner household is €65, and a two-earner household spends on average €144 per week (Eurostat, Final consumption expenditure of households by consumption purpose (coicop 3 digit), 2020). An increase of food spending by 4 to 10% would amount to €2.6 to €6.5 for a single-earner household, and to €5.76 to €14.4 for a two-earner household. Being conservative, we considered the lower part of the range, to avoid a bias against the ban. The price hike for consumers would necessarily be higher in the reference scenario (ban) than in the scenarios with lower reduction achieved by a tax. We consider a €4 cost for the ban scenario (weekly increase in food expenditure for the household), and three possible levels to build the taxation scenarios: €1, €2 and €3. While it is almost impossible to attach certainty to these cost levels, these levels seek to provide realistic scenarios for consumers.

Regarding the possible earmarking of tax revenue, we considered programs dedicated to public health, the environment and support to farmers, opposed to a flow in the government budget. The pre-experimental information provided examples of earmarking. For health programs, the examples were the monitoring of food contamination by pesticides, financing research on the impact of pesticides on our health and limiting pesticide-related harm on public health. For environmental programs, examples were related to the reduction of water and soil pollution and contamination, and biodiversity loss caused by glyphosate and pesticide use. For additional support to farmers, we highlighted the *additionality*, given that any governmental policy to phase out glyphosate already includes such a budget. This additional support would be spent on supporting farmers in their transition to a sustainable farming sector, through training, advisors, low-cost loans, insurance programs, and so on. A tax revenue flowing in the government general budget could be used on priority areas, that could be on pension programs, health, education, defense, transport, industry and employment. This revenue could also be spent to decrease another tax so as not to raise another tax such as income taxes (i.e., environmental fiscal reform [^[[12]](#endnote-12)^]).

*On the design of the experiment*

The software program NGene was used to develop the experimental design of the DCE. The attribute levels for the different scenarios were chosen based on a Bayesian D-optimal efficient design produced using NGene 1.1., with 12 rows divided into two blocks [^[[13]](#endnote-13)^]. Efficient designs are often illustrated to show statistical superiority compared to orthogonal designs, as they maximize information available by taking preliminary data into account on the preferences of the target group [^[[14]](#endnote-14)^]. The priors were chosen based on the literature and the results from the pilot study conducted with Amazon M-Turk. The order in which the choice tasks were presented to the respondent was randomized.

The following table summarizes the 12 choice tasks, grouped in the two sets of six tasks.

***Table S1. Details of the 12 choices tasks, divided in two sets of 6 choices (Choice tasks 1 to 6, and 7 to 12)***

| **CHOICE TASK 1** | **Option 1**  Ban on the use of glyphosate | **Option 2**  Tax on the use of glyphosate | **Option 3**  Tax on the use of glyphosate |
| --- | --- | --- | --- |
| Reduction in farmers’ use of glyphosate (in %) | 100 % | 75 % | 85 % |
| Increase in price of weekly shopping basket of food | + €4 per week | + €2 per week | + €3 per week |
| How the government spends the revenue from the tax on the use of glyphosate | *There is no tax revenue due to ban on glyphosate* | Environmental programmes | Flows into the government’s general budget |

| **CHOICE TASK 2** | **Option 1**  Ban on the use of glyphosate | **Option 2**  Tax on the use of glyphosate | **Option 3**  Tax on the use of glyphosate |
| --- | --- | --- | --- |
| Reduction in farmers’ use of glyphosate (in %) | 100 % | 85 % | 95 % |
| Increase in price of weekly shopping basket of food | + €4 per week | + €1 per week | + €2 per week |
| How the government spends the revenue from the tax on the use of glyphosate | *There is no tax revenue due to ban on glyphosate* | Flows into the government’s general budget | Additional support to farmers |

| **CHOICE TASK 3** | **Option 1**  Ban on the use of glyphosate | **Option 2**  Tax on the use of glyphosate | **Option 3**  Tax on the use of glyphosate |
| --- | --- | --- | --- |
| Reduction in farmers’ use of glyphosate (in %) | 100 % | 75 % | 95 % |
| Increase in price of weekly shopping basket of food | + €4 per week | + €1 per week | + €3 per week |
| How the government spends the revenue from the tax on the use of glyphosate | *There is no tax revenue due to ban on glyphosate* | Flows into the government’s general budget | Environmental programmes |

| **CHOICE TASK 4** | **Option 1**  Ban on the use of glyphosate | **Option 2**  Tax on the use of glyphosate | **Option 3**  Tax on the use of glyphosate |
| --- | --- | --- | --- |
| Reduction in farmers’ use of glyphosate (in %) | 100 % | 95 % | 85 % |
| Increase in price of weekly shopping basket of food | + €4 per week | + €3 per week | + €1 per week |
| How the government spends the revenue from the tax on the use of glyphosate | *There is no tax revenue due to ban on glyphosate* | Health programmes | Environmental programmes |

| **CHOICE TASK 5** | **Option 1**  Ban on the use of glyphosate | **Option 2**  Tax on the use of glyphosate | **Option 3**  Tax on the use of glyphosate |
| --- | --- | --- | --- |
| Reduction in farmers’ use of glyphosate (in %) | 100 % | 95 % | 75 % |
| Increase in price of weekly shopping basket of food | + €4 per week | + €2 per week | + €1 per week |
| How the government spends the revenue from the tax on the use of glyphosate | *There is no tax revenue due to ban on glyphosate* | Health programmes | Environmental programmes |

| **CHOICE TASK 6** | **Option 1**  Ban on the use of glyphosate | **Option 2**  Tax on the use of glyphosate | **Option 3**  Tax on the use of glyphosate |
| --- | --- | --- | --- |
| Reduction in farmers’ use of glyphosate (in %) | 100 % | 85 % | 75 % |
| Increase in price of weekly shopping basket of food | + €4 per week | + €3 per week | + €2 per week |
| How the government spends the revenue from the tax on the use of glyphosate | *There is no tax revenue due to ban on glyphosate* | Environmental programmes | Health programmes |

| **CHOICE TASK 7** | **Option 1**  Ban on the use of glyphosate | **Option 2**  Tax on the use of glyphosate | **Option 3**  Tax on the use of glyphosate |
| --- | --- | --- | --- |
| Reduction in farmers’ use of glyphosate (in %) | 100 % | 85 % | 85 % |
| Increase in price of weekly shopping basket of food | + €4 per week | + €2 per week | + €2 per week |
| How the government spends the revenue from the tax on the use of glyphosate | *There is no tax revenue due to ban on glyphosate* | Health programmes | Additional support to farmers |

| **CHOICE TASK 8** | **Option 1**  Ban on the use of glyphosate | **Option 2**  Tax on the use of glyphosate | **Option 3**  Tax on the use of glyphosate |
| --- | --- | --- | --- |
| Reduction in farmers’ use of glyphosate (in %) | 100 % | 95 % | 75 % |
| Increase in price of weekly shopping basket of food | + €4 per week | + €3 per week | + €1 per week |
| How the government spends the revenue from the tax on the use of glyphosate | *There is no tax revenue due to ban on glyphosate* | Flows into the government’s general budget | Health programmes |

| **CHOICE TASK 9** | **Option 1**  Ban on the use of glyphosate | **Option 2**  Tax on the use of glyphosate | **Option 3**  Tax on the use of glyphosate |
| --- | --- | --- | --- |
| Reduction in farmers’ use of glyphosate (in %) | 100 % | 75 % | 85 % |
| Increase in price of weekly shopping basket of food | + €4 per week | + €2 per week | + €3 per week |
| How the government spends the revenue from the tax on the use of glyphosate | *There is no tax revenue due to ban on glyphosate* | Additional support to farmers | Environmental programmes |

| **CHOICE TASK 10** | **Option 1**  Ban on the use of glyphosate | **Option 2**  Tax on the use of glyphosate | **Option 3**  Tax on the use of glyphosate |
| --- | --- | --- | --- |
| Reduction in farmers’ use of glyphosate (in %) | 100 % | 85 % | 95 % |
| Increase in price of weekly shopping basket of food | + €4 per week | + €1 per week | + €2 per week |
| How the government spends the revenue from the tax on the use of glyphosate | *There is no tax revenue due to ban on glyphosate* | Additional support to farmers | Health programmes |

| **CHOICE TASK 11** | **Option 1**  Ban on the use of glyphosate | **Option 2**  Tax on the use of glyphosate | **Option 3**  Tax on the use of glyphosate |
| --- | --- | --- | --- |
| Reduction in farmers’ use of glyphosate (in %) | 100 % | 75 % | 95 % |
| Increase in price of weekly shopping basket of food | + €4 per week | + €1 per week | + €3 per week |
| How the government spends the revenue from the tax on the use of glyphosate | *There is no tax revenue due to ban on glyphosate* | Environmental programmes | Flows into the government’s general budget |

| **CHOICE TASK 12** | **Option 1**  Ban on the use of glyphosate | **Option 2**  Tax on the use of glyphosate | **Option 3**  Tax on the use of glyphosate |
| --- | --- | --- | --- |
| Reduction in farmers’ use of glyphosate (in %) | 100 % | 95 % | 75 % |
| Increase in price of weekly shopping basket of food | + €4 per week | + €3 per week | + €1 per week |
| How the government spends the revenue from the tax on the use of glyphosate | *There is no tax revenue due to ban on glyphosate* | Additional support to farmers | Flows into the government’s general budget |

*On the conduct of the experiment*

All contact and questionnaire administration procedures were completed electronically and participants' anonymity was guaranteed. The survey took on average a total of 10 minutes to complete. The questions concerning the respondents' personal situation and opinions regarding environmental sensitivity - further detailed below, the (mandatory) presentation of two videos, a slide, a ranking question, the six choice tasks, and post-experimental questions relating to how clear the survey was. All respondents received the same information before beginning the DCE, and the surveys were translated into Dutch, French, German, Italian and Spanish. As research has shown that language and grammatical tense can impact economic behavior [^[[15]](#endnote-15)^], particular care was taken to ensure that the information provided in each language was as interchangeable as possible. The information in the videos was presented through subtitles in the respective language of the respondent, and therefore also in either Dutch, French, German, Italian or Spanish.

*Sample representativeness*

The survey targeted the main person responsible for purchasing food in the household (the primary grocery shopper aged 18-70 years-old). In total, 2 050 household representatives took part in the study. Each country's sample was representative of the national population at the household level on the following quotas: age and gender of the primary grocery shopper, geographical location (region; rural vs. urban) and level of education. Sample size for each country was chosen in proportion with the national population (Eurostat data, 2021), with a minimum sample size of 150 respondents per country in order to perform any meaningful econometric analysis at the country level [50]: the over-representativeness of Belgium is due to this minimum requirement. A robustness check was performed by dropping Belgium from the sample. Results remained similar and illustrated that Belgium did not skew the results despite its over-representativeness. Table S2 illustrates the sample size and the total population (in 1 000) in each country.

***Table S2. Sample size adjusted from relative population ratios***

|  | **Belgium** | **France** | **Germany** | **Italy** | **Spain** |
| --- | --- | --- | --- | --- | --- |
| Population (in 1 000) | 11 455.5 | 67 012.9 | 83 019.2 | 60 359.5 | 49 937.1 |
| Proportion | 4% | 25% | 31% | 22.5% | 17.5% |
| Sample size per country  (N [total]= 2 050) | 150 | 500 | 600 | 450 | 450 |

The average age of respondents was 45, with ages varying from 18 to 70 years old. The gender distribution was almost exactly 50/50, while the distribution of educational attainment was representative of the general population. For each country, the respondents' educational attainment was coded and divided into three main levels: low, medium and high. Low referred to no degree, lower secondary education of three years (both general and technical); medium referred to upper secondary education of six years (general/technical or professional vocational education and training) and finally, higher education referring to tertiary level education (such as bachelor's level degrees, postgraduate, master's degrees and doctorate level degrees). In our sample, the majority of respondents had attained a medium level education, which corresponds to EU-wide statistics on educational attainment across a similarly distributed age group (Eurostat, educational attainment statistics, 2020).

In order to capture the purchasing power of respondents, they were asked how much their household spent on food per week: this amounted to an average of €94.13 per week. Eurostat data shows that in single person households average household spending on food ranges from €55.18 to €76.125 per week, while spending on food in two-earner households with two children ranges from €112.93 to €166.06 per week, with lowest food consumption being Spain, to highest being in France; Germany, Italy and Belgium fall in the middle of the five countries in our sample, in said order (Eurostat. Final consumption expenditure of households by consumption purpose (coicop 3 digit), 2020). The average household size in our sample equaled 2.57 while the EU average household size is 2.3 (Eurostat, Household composition statistics, 2020).

***Table S3. Descriptive statistics of sample***

| **Variable** | **Mean** | **SD** | **%** |
| --- | --- | --- | --- |
| **Country surveyed** |  |  |  |
| Belgium |  |  | 7 |
| France |  |  | 25 |
| Germany |  |  | 29 |
| Italy |  |  | 22 |
| Spain |  |  | 17 |
| **Age** | 44.5 | 14.42 |  |
| **Gender (F=1)** |  |  | 50.24 |
| **Education** |  |  |  |
| Low |  |  | 30.94 |
| Medium |  |  | 42.16 |
| High |  |  | 26.63 |
| **Living in a rural area** |  |  | 12.57 |
| **Food expenditure per week** | €94.13 | 44.48 |  |
| **Household size** | 2.57 | 1.26 |  |
| **Frequency of organic food consumption** |  |  |  |
| (Almost) never |  |  | 29.27 |
| 1-3 times a month |  |  | 20.20 |
| 1 to several times a week |  |  | 35.02 |
| (Almost) every day |  |  | 15.51 |
| **Had already heard of glyphosate** |  |  | 65.51 |
| **Share of agreement on whether:** |  |  |  |
| Glyphosate is harmful to public health |  |  | 81.09 |
| Glyphosate is harmful to the environment |  |  | 82.65 |
| Glyphosate use should be taxed |  |  | 63.9 |
| Glyphosate use should be banned |  |  | 75.5 |
| **Share of agreement (if never heard of glyphosate) on whether:** |  |  |  |
| Pesticides are harmful to public health |  |  | 74.83 |
| Pesticides are harmful to the environment |  |  | 78.79 |
| Pesticide use should be taxed |  |  | 48.38 |
| Pesticide use should be banned |  |  | 56.72 |

Data analysis: methodological details

As the DCE presented respondents with three alternatives at a time (including the *status quo* scenario), the model featured two constants for the first and second alternative with respect to the *status quo*. The *status quo* alternative is perfectly confounded with the baseline of the attribute related to how the government would be spending the money collected *via* the tax. As a result, three dummy variables are introduced in the model to capture the effect of ‘environmental programs’, ‘health programs’ and ‘additional support for farmers’ with respect to the *status quo* and ‘unspecified revenue allocation by the government’. The attributes related to ‘increase in prices as a result of the ban’ and ‘reduction in farmers’ use of glyphosate’ were specified as continuous variables. Econometric analysis was performed using the Apollo package for R [^[[16]](#endnote-16)^,^[[17]](#endnote-17)^].

*Latent class mixed logit analysis*

The bulk of the empirical work consisted in analyzing the stated choices of respondents across the countries considered. This is achieved by modeling the choices between the three options presented in each scenario. Given the divisive nature of the alternatives presented to the respondents, leading to high levels of heterogeneity in preferences, we used Latent Class Mixed Logit Models (LC-MXL). There are precedents for using latent class models in the literature in order to account for the fact that respondents might show extreme preferences for the different options proposed [^[[18]](#endnote-18)^,^[[19]](#endnote-19)^]. While the latent class structure assumes discrete heterogeneity in preferences (the model predicts the likelihood that respondents belong to a given class), the mixed logit model component assumes continuous heterogeneity in preferences for some of the attributes considered. Preliminary analysis showed that a large number of respondents were likely to either always choose the status-quo alternative or always choose the tax options, which illustrates the strong divide between respondents and justifies the use of a latent class structure. Continuous heterogeneity in preferences is only assumed for the price attribute, which is specified as negative μ-shifted log-normally distributed [^[[20]](#endnote-20)^] in order to account for the fact that different respondents might have a different marginal utility of income within each latent class.

We assume that the utility that respondent *n* derives from choosing alternative *i* in choice situation t can be decomposed into a deterministic part on the one hand and a stochastic part on the other hand, as described in Equation (1) below:

$$U_{nit} = {{\beta{'x}_{nit} + \epsilon_{nit}}} \left( 1 \right)$$

where $\beta$ is a vector of parameters to be estimated, $x_{nit}$ is a vector of attributes and $\epsilon_{nit}$is a type I extreme value distributed error term. These assumptions give rise to the multinomial logit model, for which the probability of the sequence of choices made by individual n (formalized as $y_{n}= i_{n1}, i_{n2}, ...,i_{n6}$) corresponds to (see [^[[21]](#endnote-21)^])

$$Pr\left( y_{n}|x_{n} \right)=\prod_{t\in T_{n}} \frac{exp \left( \beta^{'}x_{nit} \right)}{\sum_{j\in J} exp \left( \beta^{'}x_{nij} \right)} \left( 2 \right)$$

As previously stated, the MNL model is restrictive in the sense that it does not account for discrete or random heterogeneity in preference, essentially assuming that all the respondents have the same sensitivities for each one of the attributes considered unless interaction variables related to the observed characteristics of the respondents are introduced in the model. Random heterogeneity in preferences can be introduced by estimating a mixed logit model where the parameters related to the sensitivity for a given set of attributes can be specified as randomly distributed. Let $f(\theta_{n}|\Omega)$ be the joint density over a given set of K parameters $\beta_{n1}, \beta_{n2}, ...,\beta_{nK}$ where $\theta_{n}$ is a vector of random parameters and $\Omega$ the parameters of the distributions. The unconditional probability of observing the sequence of choices made by individual n now corresponds to the integral over the multinomial logit formula for all possible values of $\theta$. The model does not have a closed-form solution so the integral found in equation (3) is solved through simulation:

$$Pr\left( y_{n}|x_{n},\Omega\right)=\int\prod_{t\in T_{n}} \frac{exp \left( \beta^{'}x_{nit} \right)}{\sum_{j\in J} exp \left( \beta^{'}x_{nij} \right)}f\left( \Omega\right)d\left( \theta_{n} \right) \left( 3 \right)$$

In the final model, the only parameters in each class which are assumed to be randomly distributed are the parameters related to price sensitivity and labeled as $\beta_{Price A}$ and $\beta_{Price B}$. As previously stated, sensitivity for the price attribute is assumed to follow a negative μ-shifted log-normal distribution. It comes that:

$\beta_{Price A} = -exp(\mu_{Price A})-exp(\mu_{Price A}+ \sigma_{Price A}\zeta_{Price A})$

and $\beta_{Price B} = -exp(\mu_{Price B})-exp(\mu_{Price B}+ \sigma_{Price B}\zeta_{Price B})$

where $\mu_{Price A}$, $\mu_{Price B}$, $\sigma_{Price A}$ and $\sigma_{Price B}$ are parameters to be estimated and $\zeta_{Price A}$ and $\zeta_{Price B}$ are random draws from standard normal distributions.

Finally, we allow for discrete heterogeneity in preferences by the means of a latent class allocation model over the mixed logit model structure. As previously discussed in the paper, we assume two latent classes, one which captures pro-ban and one which captures anti-ban in order to facilitate the interpretation of the results and given the strong divide in responses reported above. The parameters in the two classes are different. Whether a respondent belongs to the pro-ban class or the anti-ban class is unobserved by the analyst, but it can be estimated up to a probability. The probability $\omega$ for a respondent to belong to a given class is given by a multinomial logit probability. In the context of this paper, the probability to belong to class A corresponds to:

$$\omega_{A}=\frac{exp(C_{A}z_{n})}{1+ exp(C_{A}z_{n})}$$

where $C$ is a vector of parameters to be estimated and $z_{n}$a vector of covariates related to the characteristics of the respondents. The log-likelihood of the LC-MXL model featured in this paper hence corresponds to:

$$Pr\left( y_{n}|x_{n},\omega_{n},\Omega\right)=\sum_{q\in Q} \omega_{nq}\int\prod_{t\in T_{n}} \frac{exp\left( \beta^{'}x_{nit} \right)}{\sum_{j\in J} exp \left( \beta^{'}x_{nij} \right)}f\left( \Omega\right)d(\theta_{n})$$

The LC-MIXL model featured in this paper has been estimated using 10,000 draws. Estimations were performed on the Myria HPC system (CRIANN, Normandy, France).**Supplementary Text References**

1. [] Agreste. (2019) Statistique agricole annuelle 2018-2019. Retrieved from <https://agreste.agriculture.gouv.fr/agreste-web/disaron/Chd2011/detail/> on January 23, 2023. [↑](#endnote-ref-1)
2. [] Johnston, R.J., Boyle, K.J., Adamowicz, W., Bennett, J., Brouwer, R., Cameron, T.A., Hanemann, W.M., Hanley, N., Scarpa, R., Tourangeau, R., Vossler, C.A. (2017). “Contemporary guidance for stated preference studies.”, Journal of the Association of Environmental and Resource Economists., 4(2). pp. 319-405. [↑](#endnote-ref-2)
3. [] Lew, D. K., Layton, D. F., Rowe, R. D. (2010). Valuing enhancements to endangered species protection under alternative baseline futures: the case of the Steller sea lion. *Marine Resource Economics*, *25*(2), 133-154. [↑](#endnote-ref-3)
4. [] Kontoleon, A., Yabe, M. (2003). Assessing the impacts of alternative ‘opt-out’formats in choice experiment studies: consumer preferences for genetically modified content and production information in food. *Journal of Agricultural policy and Resources*, *5*(1), 1-43. [↑](#endnote-ref-4)
5. [] Hernández, K., Pérez, F. (2021) Contaminación por glifosato en el medio acuático. Ecologistas en Acción. Retrieved from <https://www.ecologistasenaccion.org/wp-content/uploads/2021/11/Informe-glifosato-2021.pdf> on January 23, 2023. [↑](#endnote-ref-5)
6. [] World Health Organization. (2015) IARC monographs volume 112: evaluation of five organophosphate insecticides and herbicides. Retrieved from <https://www.iarc.who.int/wp-content/uploads/2018/07/MonographVolume112-1.pdf> on January 23, 2023. [↑](#endnote-ref-6)
7. [] European Parliament. (2017) Monsanto papers and glyphosate: Lessons for the EU. Retrieved from <https://www.europarl.europa.eu/news/en/press-room/20171009IPR85652/monsanto-papers-and-glyphosate-lessons-for-the-eu> on January 23, 2023. [↑](#endnote-ref-7)
8. [] Caussade, S., de Dios Ortúzar, J., Rizzi, L. I., Hensher, D. A. (2005). Assessing the influence of design dimensions on stated choice experiment estimates. *Transportation research part B: Methodological*, *39*(7), 621-640. [↑](#endnote-ref-8)
9. [] Hanley, N., Wright, R. E., Koop, G. (2002). Modelling recreation demand using choice experiments: climbing in Scotland. *Environmental and resource Economics*, *22*(3), 449-466. [↑](#endnote-ref-9)
10. [] Fogliatto, S., Ferrero, A., Vidotto, F. (2020). Current and future scenarios of glyphosate use in Europe: Are there alternatives?. *Advances in agronomy*, *163*, 219-278. [↑](#endnote-ref-10)
11. [|] Porter, M. E., Van der Linde, C. (1995). Toward a new conception of the environment-competitiveness relationship. *Journal of economic perspectives*, *9*(4), 97-118. [↑](#endnote-ref-11)
12. [] Bach, S., Kohlhaas, M., Meyer, B., Praetorius, B., Welsch, H. (2002). The effects of environmental fiscal reform in Germany: a simulation study. *Energy policy*, *30*(9), 803-811. [↑](#endnote-ref-12)
13. [] Choice Metrics (2012). Ngene 1.1. 1 user manual & reference guide. Sydney, Australia: ChoiceMetrics, 19, 20. [↑](#endnote-ref-13)
14. [] Rose, J. M., Bliemer, M. C., Hensher, D. A., Collins, A. T. (2008). Designing efficient stated choice experiments in the presence of reference alternatives. Transportation Research Part B: Methodological, 42(4), 395-406. [↑](#endnote-ref-14)
15. [] Chen, M. K. (2013). The effect of language on economic behavior: Evidence from savings rates, health behaviors, and retirement assets. American Economic Review, 103(2), 690-731. [↑](#endnote-ref-15)
16. [] Hess, S., Palma, D. (2019). Apollo: a flexible, powerful and customisable freeware package for choice model estimation and application. Journal of Choice Modelling 32. [↑](#endnote-ref-16)
17. [] R Core Team (2022). R: A Language and Environment for Statistical Computing. R Foundation for Statistical Computing, Vienna, Austria. [https://www.R-project.org/](https://www.r-project.org/). [↑](#endnote-ref-17)
18. [] Meyerhoff, J., Bartczak, A., Liebe, U. (2012). Protester or non‐protester: a binary state? On the use (and non‐use) of latent class models to analyse protesting in economic valuation. Australian Journal of Agricultural and Resource Economics, 56(3), 438-454. [↑](#endnote-ref-18)
19. [] Hess, S., Lancsar, E., Mariel, P., Meyerhoff, J., Song, F., van den Broek-Altenburg, E., ... Zuidgeest, M. H. (2022). The path towards herd immunity: Predicting COVID-19 vaccination uptake through results from a stated choice study across six continents. Social Science & Medicine, 298, 114800. [↑](#endnote-ref-19)
20. [] Crastes dit Sourd, R. (2021) A New Shifted Log-Normal Distribution for Mitigating 'exploding' Implicit Prices in Mixed Multinomial Logit Models. Leeds University Business School Working Paper, Available at SSRN: https://ssrn.com/abstract=3878952 or http://dx.doi.org/10.2139/ssrn.3878952 [↑](#endnote-ref-20)
21. [] McFadden, D. (1974) Conditional logit analysis of qualitative choice behavior, in D. McFadden and P. Zarembka (Ed.), Frontiers in Econometrics, Academic Press, New York, pp. 105-142 [↑](#endnote-ref-21)
